# Newborn low birth weight: do socio-economic inequality still persist in India?

**DOI:** 10.1186/s12887-021-02988-3

**Published:** 2021-11-19

**Authors:** Prem Shankar Mishra, Debashree Sinha, Pradeep Kumar, Shobhit Srivastava, Rahul Bawankule

**Affiliations:** 1 Population Research Centre, Institute for Social and Economic Change, Bengaluru, Karnataka 560072 India; 2 Department of Development Studies, International Institute for Population Sciences, Mumbai, Maharashtra 400088 India; 3 Department of Mathematical Demography & Statistics, International Institute for Population Sciences, Mumbai, Maharashtra 400088 India; 4 Department of Public Health & Mortality Studies, International Institute for Population Sciences, Mumbai, Maharashtra 400088 India

**Keywords:** Low birth weight, Socio-economic inequality, Concentration index, Decomposition, India

## Abstract

**Background:**

The incidence of preterm birth and subsequent low birth weight (LBW) are vital global public health issues. It contributes to high infant and child mortality in the early stages of life and later on in adult life; it increases the risk for non-communicable diseases. The study aims to understand the socio-economic status-related inequality for LBW among children in India. It hypothesises that there is no association between the socio-economic status of the household and the newborn’s LBW in India.

**Methods:**

The study utilised data from the fourth round of the National Family Health Survey, a national representative cross-sectional survey conducted in 2015-16 (*N* = 127,141). The concentration index (CCI) and the concentration curve (CC) measured socio-economic inequality in low birth status among newborns. Wagstaff decomposition further analysed key contributors in CCI by segregating significant covariates.

**Results:**

About 18.2% of children had low birth weight status. The value of concentration was − 0.05 representing that low birth weight status is concentrated among children from lower socio-economic status. Further, the wealth quintile explained 76.6% of the SES related inequality followed by regions of India (− 44%) and the educational status of mothers (43.4%) for LBW among children in India. Additionally, the body mass index of the women (28.4%), ante-natal care (20.8%) and residential status (− 15.7%) explained SES related inequality for LBW among children in India.

**Conclusion:**

Adequate attention should be given to the mother’s nutritional status. Awareness of education and usage of health services during pregnancy should be promoted. Further, there is a need to improve the coverage and awareness of the ante-natal care (ANC) program. In such cases, the role of the health workers is of utmost importance. Programs on maternal health services can be merged with maternal nutrition to bring about an overall decline in the LBW of children in India.

**Supplementary Information:**

The online version contains supplementary material available at 10.1186/s12887-021-02988-3.

## Background

The incidence of preterm birth and subsequent low birth weight (LBW) are vital global public health issues [1]. It contributes to a high infant and child mortality in the early stages of life and later on in adult life; it increases the risk for non-communicable diseases [1–3]. The incidence of LBW is defined as the proportion of newborns weighing less than 2500 g (< 5.5 lbs), regardless of gestational age [2]. Worldwide, in 2013, an estimation showed that nearly 22 million newborns (nearly 16% of all babies born globally) were LBW [2]. The majority of LBW occurs in low and middle-income countries, and it occurs to the marginalized and vulnerable sub-population groups [1, 4]. The goal is to achieve a 30% reduction in the number of infants born with a weight less than 2500 g by 2025 [2], however, a lack of policy consistency and implementation strategy across low and middle-income countries are unable to achieve it. Moreover, regional variations in LBW are found uneven, and some of the regions like South Asia, Sub-Saharan Africa are facing higher severity in newborn LBW [1, 4]. Further, regional level estimates show that the occurrence of LBW in South Asia is 28 and 13% in Sub-Saharan Africa, respectively [2]. Within the southern region, India contributes the highest infant LBW [1, 5, 6]; therefore, it becomes important to understand the co-existing determinants that affect LBW in India and across regions.

LBW is one of the most important indicators to measure the socio-economic development of the community. The mother’s socio-economic characteristics are directly linked to the child’s health and well-being. Studies have shown that mother’s education, knowledge and media exposure are significantly linked to reducing LBW in low and middle-income countries, including India [4, 5, 7]. In India, women have been historically deprived on multiple socio-political and economic grounds that have further put their children at high risk, such as preterm birth and LBW [5, 8–10]. Furthermore, there is a substantial variation in the prevalence of LBW across different socio-economic groups and regions in India [5, 11]. However, the majority of LBW is found among the most deprived communities [9, 12–14]. Although LBW is the outcome of multi-faceted risk factors yet very few studies have tried to understand it. Further, low resource setting areas are also vulnerable to the high occurrence of LBW [15].

Since the mother’s health and nutritious characteristics play a significant role in the outcomes of preterm birth and LBW among their children, it is, therefore, essential to provide proper health, diet, and nutritional care to mothers during pregnancy [1, 2, 7, 16]. The mother’s nutritional deficiency and poor healthcare services put the baby at risk, such as preterm birth or LBW. Further, providing reproductive health services such as contraception to delay age at first pregnancy and to increase intervals between births can reduce the chance of delivering an LBW newborn [8, 17, 18]. Moreover, for a healthy baby, mothers need better health care services (full antenatal care), proper nutrition, and a clean environment [1, 6, 7]. Smoking and drinking alcohol among women can further create a risk for LBW [19].

The incidence of newborn LBW is an important predictor of newborn health, growth, cognitive development, and survival [2]. The mother’s continuity of care may reduce newborn LBW. Although there are several consecutive interventions to mother and child to enhance child health and survivorship, however, in India, lack in pre-pregnancy interventions, full antenatal care interventions, and interventions at the community level contribute to adverse outcomes of a newborn [5, 7, 15]. Nevertheless, occurring LBW among newborn babies constitutes several other problems that have been recognised as poor maternal and child care in the community, poor referral system, poor health infrastructure, home-based care, and lack of surveillance and data management regarding it [1, 20–22].

In this way, there are many predisposing risk factors that influence LBW in India. Very few studies have compared the prevalence of LBW across different gradients of women and children, such as socio-economically, demographically, and environmentally in India and across regions. A dearth of literature is found to understand the multiple determinants that are pervasive to LBW in India and across regions. Therefore, the study aims to understand the socio-economic status-related inequality for LBW among children in India. The study further hypothesises that there is no association between the socio-economic status of the household and the newborn’s LBW in India.

## Methods

We used data from the fourth round of the National Family Health Survey (NFHS), a nationally representative cross-sectional survey conducted in 2015–16. The International Institute for Population Sciences (IIPS) in Mumbai has conducted four rounds of NFHS surveys under the aegis of the Indian Ministry of Health and Family Welfare (MoHFW). The NFHS-4’s main goal was to offer vital statistics on health and family welfare, as well as data on emerging difficulties in these areas. As a result, the survey collects data on population health and nutrition for men, women, and children in all 36 states and union territories of India. To obtain estimates for India as a whole and its states, the NFHS-4 used a two-stage stratified sampling approach. The details can be obtained from elsewhere [23]. With a response rate of 98%, 97%, and 92%, the survey collected data from 601,509 households, 699,686 women, and 112,122 men.

For the newborn’s birth weight, a question was asked to the mothers ‘Was (NAME) weighed at birth? If the response was yes, further, ‘How much did (NAME) weigh? The responses were recorded as weight in kilograms from card and kilograms from recall. Among 259,627 sampled children, 57,451 children were not weighed at birth, and 8818 were “don’t know” cases. After excluding children whose birth weight data were not available, finally, we had 193,358 children with LBW data. In order to obtain better estimates for the body mass index (BMI), the study excluded women who were presently pregnant and women who had given birth in the previous 2 months. The NFHS-4 gathered data on the three youngest children from the birth history who were born within the survey’s reference period. To eliminate recall bias, we only included the most recent birth in the 5 years before to the survey. Also, for the second and third most recent births in history, information on Antenatal Care (ANC) associated characteristics was not accessible. As a result, we had a total of 127,141 index children in our analytical sample. Table S1 contains the strobe guidelines (supplementary file).

### Outcome variable

All newborns weighing less than 2.5 kg (< 2500 g) at the time of birth was considered low birth weight (LBW) children. The outcome variable was dichotomous: 1 ‘yes’ if newborns weighing less than 2.5 kg at the time of birth and 0 ‘no,’ otherwise [12, 24–26].

### Exposure variables

Based on the literature, we identified potential risk factors of LBW and included them as exposure variables in the analysis [1, 2, 5, 9, 16, 22–24]. The exposure variables included age of women at index birth (≤19, 20-24, 25-29, 30-35 and 35+ years), body mass index (underweight: ≤18.5 kg/m^2^, normal: 18.5 to < 24.99 kg/m^2^ and overweight/obesity: ≥25.0 kg/m^2^) [27], and antenatal care (no, partial, and full). The study created a composite variable ‘birth order and interval’ using the information on the birth order number and preceding birth interval from the index child. Further, we categorised it as ‘first birth order’, ‘second and third birth order and birth interval lesser than 24 months (2-3 & < 24)’, ‘second and third birth order and birth interval greater than 23 months (2–3 & > 23)’ ‘four and above birth order and birth interval lesser than 24 months (≥ 4 & < 24)’, ‘four and above birth order and birth interval greater than 23 months (≥ 4 & > 23)’. Other predictors were education (no education, primary, secondary, and higher), caste (scheduled caste (SC), scheduled tribe (ST), other backward class (OBC), and others) [28], and religion (Hindu, Muslim, and others (including Christian, Sikh, Buddhist/Neo-Buddhist, Jain, Jewish, Parsi/Zoroastrian, no religion, and others). A household’s wealth index was calculated in the survey by combining household amenities, assets, and durables and characterising households in a range varying from the poorest to the richest, corresponding to wealth quintiles ranging from the lowest to the highest [29]. Further, the place of residence (urban and rural), and sex of the child (male and female) were included. Geographical regions were coded as North, Central, East, Northeast, West, and South. The source of drinking water was coded into two categories: unimproved and improved [30]. We included public taps, piped water, tube wells, boreholes, standpipes, protected dug wells and springs, rainwater, and community reverse osmosis (RO) plants as an improved source of drinking water. Similarly, the type of toilet facility was coded as unimproved and improved [30]. The flush/pour flush toilets to piped sewer systems, septic tanks, and pit latrines; ventilated improved pit (VIP)/biogas latrines; pit latrines with slabs; and twin pit/composting toilets were included as an improved type of toilet facility. The type of cooking fuel was grouped as unclean and clean [30]. We termed cooking fuel as clean if households used electricity, LPG/natural gas, biogas for cooking purposes. Finally,a complete ANC was defined as a mother who had four or more antenatal visits, at least two tetanus toxoid shots, and took iron and folic acid pills or syrup for 100 days or more during their most recent live birth in the 5 years before to the survey.

### Statistical analysis

The distribution of the study population was depicted using descriptive statistics. The factors linked with the outcome variable were also identified using bivariate and multivariate logistic regression analysis. To further understand the relationship (significant or not significant) between outcome variables and predictors, a Chi-square test was used.

### Concentration index

The wealth quintile was the most important factor in determining a family’s economic state. To undertake decomposition analysis and produce the concentration index, we first calculated the wealth score (CCI). The children were then graded and divided into five equal categories, each having 20% of the children, based on their wealth ratings [31, 32]. To quantify socio-economic inequality in LBW babies in India, the concentration index and concentration curve (CC) were calculated [28, 33, 34]. The concentration index is calculated by dividing the area between the concentration curve and the line of equality by twice the weighted covariance between the result and fractional rank in the wealth distribution divided by the variable mean [31, 32].

The concentration index can be written as follows:

$$\boldsymbol{C}=\frac{\mathbf{2}}{\boldsymbol{\mu}}\boldsymbol{\operatorname{cov}}\left({\boldsymbol{y}}_{\boldsymbol{i},}{\boldsymbol{R}}_{\boldsymbol{i}}\right)$$

Where C is the concentration index; *y*_*i*_ is the outcome variable index; ***R*** is the fractional rank of individual ***i*** in the distribution of socio-economic position; ***μ*** is the mean of the outcome variable of the sample, and ***cov*** denotes the covariance [35]. The index value lies between − 1 to + 1.

The concentration index takes a negative value if the curve is above the line of equality, suggesting a disproportionate concentration of inequality among the poor (pro-rich) [31, 32]. If the curve falls below the line of equality, the concentration index is positive, suggesting that inequality is concentrated disproportionally among the rich (pro-poor) [31, 32]. The concentration index is zero when there is no socioeconomic disparity [31, 32]. The CI value indicates the degree of socioeconomic disparity. The greater the absolute value, the more inequities there are.

### Decomposition of the concentration index

Wagstaff decomposition methodology was used to decompose the concentration index [35]. The concentration index could be broken down into the contributions of each aspect to income disparities, according to Wagstaff’s decomposition [36]. Each contribution is determined by the health sensitivity of that socioeconomic component as well as the degree of income disparity in that factor. Based on the linear regression relationship between the outcome variable *y*_*i*_, the intercept α, the relative contribution of *x*_*ki*_ and the residual error *ε*_*i*_

$${y}_i=\alpha +\sum {\beta}_k{x}_{ki}+{\varepsilon}_i$$

Where *ε*_*i*_ is an error term, given the relationship between *y*_*i*_ and *x*_*ki*_, the CCI for y (C) can be rewritten as

$$C=\sum \left(\frac{\beta_k{\overline{x}}_k}{\mu}\right){C}_k+\frac{GC\varepsilon}{\mu }/\mu$$

Where *μ* is the mean of *y*_*i*_, ${\overline{x}}_k$, is the mean of *x*_*k*_, *β*_*k*_ is the coefficient from a linear regression of outcome variable, *C*_*k*_ is the concentration index for *x*_*k*_ (defined analogously to C, and GC_ɛ_ is the generalised concentration index for the error term (*ε*_*i*_).

Here C is the outcome of two components: First, there are the determinants, or ‘explained’ factors, which are comparable to the weighted accumulation of the regressor’s concentration indices, where one unit change in the outcome variable corresponds to one unit change in the explanatory variable [31, 32]. The explained factors indicate that the proportion of inequalities in the outcome (LBW) variable is explained by the selected explanatory factors, i.e., x_k_. Second, a residual or ‘unexplained’ factor $\left(\frac{GC\varepsilon}{\mu }/\mu \right)$, indicating the inequality in health variables that cannot be explained by selected explanatory factors across various socio-economic groups [35]. The percentage contribution column should be interpreted as the percentage contribution of factors that explain SES-related inequality in LBW among Indian children. The overall contribution is calculated using the negative and positive signs, which is dependent on the table’s sign elasticity and CCI [31, 32]. The absolute contribution is the product of elasticity and CCI. Furthermore, dividing the aggregate absolute contribution by the absolute contributions of the various variables yields the individual contribution. As a result, the elasticity and CCI define the size of the percentage contribution [33].

## Results

The percentage distribution of the study population by background characteristics is presented in Table 1. About 2.3% of women interviewed had their age at index birth of 35 years and above. Nearly 25% of women were underweight, and 18% were either overweight or obese. Only 24% of women received full ANC, whereas 6 % received no ANC. Almost 9 % of women had children with fourth birth order and birth interval of more than 24 months. About 21% of women had no education in contrast to 15% who had completed higher education. About 16%, 45% and 57% of households had no improved drinking water, no improved toilet facility and unclean cooking fuel facility, respectively.

**Table 1:** Percentage distribution of the study population by background characteristics, India, 2015–16

Background characteristics	Percentage	Sample
**Age at index birth (in years)**		
≤ 19	14.3	18,144
20–24	44.8	57,013
25–29	28.0	35,622
30–35	10.6	13,485
35+	2.3	2876
**Body mass index**		
Underweight	24.5	29,946
Normal	57.5	75,821
Overweight and Obese	18.0	21,374
**Ante-natal care**		
No	5.9	8223
Partial	70.6	91,977
Full^a^	23.6	26,941
**Birth order and interval**		
First	34.9	43,295
2–3 & < 23 months	12.8	15,263
2–3 & > 23 months	41.0	51,640
4+ & < 24 months	2.5	3643
4+ & > 24 months	8.8	13,300
**Education**		
No education	20.5	27,650
Primary	12.7	16,579
Secondary	52.4	66,108
Higher	14.5	16,804
**Caste**		
Scheduled Caste	20.5	23,074
Scheduled Tribe	9.6	22,755
Other Backward Class	42.8	49,913
Others	27.1	31,399
**Religion**		
Hindu	80.3	95,539
Muslim	14.2	17,005
Others	5.4	14,597
**Wealth index**		
Poorest	17.0	23,287
Poorer	19.8	26,643
Middle	21.1	27,214
Richer	21.9	25,895
Richest	20.2	24,102
**Residence**		
Urban	34.0	36,874
Rural	66.0	90,267
**Sex of the child**		
Male	55.5	70,380
Female	44.5	56,761
**Region**		
North	13.6	25,133
Central	20.1	30,284
East	23.9	25,385
Northeast	3.8	18,059
West	15.6	11,289
South	22.9	16,991
**Source of drinking Water**		
Not improved	15.6	20,723
Improved^b^	84.4	106,418
**Type of toilet facility**		
Not improved	44.8	57,163
Improved^c^	55.2	69,978
**Type of cooking fuel**		
Unclean	56.9	78,816
Clean^d^	43.1	48,325
**Total**	100.0	127,141

^a^Full ANC defined as mother who received four or more antenatal checks, at least two tetanus toxoid injection, and took iron and folic acid tablets or syrup for 100 days or more during their last live birth in the 5 years preceding the survey

^b^Include piped water, public taps, standpipes, tube wells, boreholes, protected dug wells and springs, rainwater, and community reverse osmosis (RO) plants

^c^Include flush/pour flush toilets to piped sewer systems, septic tanks, and pit latrines; ventilated improved pit (VIP)/biogas latrines; pit latrines with slabs; and twin pit/composting toilets

^d^Include Electricity, LPG/natural gas, biogas

The percentage distribution of LBW among children by background characteristics in India is presented in Table 2. The prevalence of LBW was high among children whose mother had an age of index birth below or equaled 19 years (19.9%). The underweight women had a higher LBW prevalence in their children (21.1%). Mother’s with no ANC reported higher LBW prevalence among their child (22.9%). The prevalence of LBW was higher among children from fourth birth order and birth interval less than 24 months (20.3%). Mother’s with no or primary education reported higher LBW prevalence among their child (19.4% and 19.8%), respectively. The prevalence of LBW among children was considerably higher in rural areas (17.8%) compared to urban areas. Female children had a higher prevalence of LBW (18.9%) than male children. Women from the northern and eastern regions of India reported a higher prevalence of LBW among their children (19.8% and 19.3%), respectively. Children from the household with not improved toilet facility had a higher prevalence of LBW (18.8%). Households with unclean cooking fuel had a higher prevalence of LBW in children (18.3%) than children in households with clean cooking fuel.

**Table 2:** Percentage distribution of low birth weight (LBW: < 2500 g) among children by background characteristics, India, 2015–16

Background characteristics	LBW	***P***-value
**Age at index birth (in Years)**		*
≤ 19	19.9	
20–24	16.9	
25–29	16.7	
30–35	17.1	
35+	18.5	
**Body mass index**		*
Underweight	21.1	
Normal	16.6	
Overweight and Obese	14.5	
**Ante-natal care**		*
No	22.9	
Partial	17.8	
Full^a^	14.5	
**Birth order and interval**		*
First	18.0	
2–3 & < 23 months	17.7	
2–3 & > 23 months	16.3	
4+ & < 24 months	20.3	
4+ & > 24 months	17.9	
**Education**		*
No education	19.4	
Primary	19.8	
Secondary	17.1	
Higher	13.3	
**Caste**		*
Scheduled Caste	18.4	
Scheduled Tribe	19.3	
Other Backward Class	17.0	
Others	16.3	
**Religion**		
Hindu	17.6	
Muslim	16.2	
Others	15.7	
**Wealth index**		*
Poorest	19.6	
Poorer	18.3	
Middle	17.6	
Richer	17.5	
Richest	14.1	
**Residence**		*
Urban	16.5	
Rural	17.8	
**Sex of the child**		*
Male	16.1	
Female	18.9	
**Region**		*
North	19.8	
Central	19.3	
East	15.7	
Northeast	13.6	
West	18.3	
South	15.9	
**Source of drinking Water**		
Not improved	17.8	
Improved^b^	17.3	
**Type of toilet facility**		*
Not improved	18.8	
Improved^c^	16.2	
**Type of cooking fuel**		*
Unclean	18.3	
Clean^d^	16.1	
**Total**	18.2	

*LBW* Low birth weight

*p < 0.05

^a^Full ANC defined as mother who received four or more antenatal checks, at least two tetanus toxoid injection, and took iron and folic acid tablets or syrup for 100 days or more during their last live birth in the 5 years preceding the survey

^b^Include piped water, public taps, standpipes, tube wells, boreholes, protected dug wells and springs, rainwater, and community reverse osmosis (RO) plants

^c^Include flush/pour flush toilets to piped sewer systems, septic tanks, and pit latrines; ventilated improved pit (VIP)/biogas latrines; pit latrines with slabs; and twin pit/composting toilets

^d^Include Electricity, LPG/natural gas, biogas

Table 3 presents the estimates from logistic regression analysis for LBW children by their background characteristics. It was found that women who gave their index birth at the age of 20-24 years had an 18% lower likelihood to have children with LBW status than women who gave birth at age 19 years or less [OR:0.82, CI:0.79-0.86]. Women with underweight as BMI status had a higher likelihood to have children with LBW status than women with normal BMI status [OR: 1.27, CI: 1.23-2.32]. Children whose mothers had full ANC had lower odds of LBW compared to those who had no ANC [OR: 0.64, CI: 0.60-0.68]. The children with 4+ birth order and birth interval as more than 23 months had a 19% lower likelihood to be born as LBW [OR:0.8, CI: 0.76-0.86] in comparison to children born as first birth order. Women with higher education status had a 27% lower likelihood to have children with LBW than women with no education [OR: 0.73, CI: 0.69-0.79]. Women from the richest wealth quintile had a 21% lower likelihood to have children with LBW than women from the poorest households [OR: 0.79, CI: 0.73-0.85]. Women from rural areas had lower odds to have LBW than children from urban areas [OR:0.89, CI: 0.86-0.93]. Female newborns had a 23% higher likelihood to have LBW status than male newborns [OR: 1.23, CI: 1.19 -1.26]. Women who were from the north-eastern region of India had a 49% lower likelihood to have LBW children than women from the northern region of India [OR:0.51, CI:0.47-0.51]. Similarly, women from households with clean cooking fuel had a 4% lower likelihood to be LBW than women from households with unclean cook fuel [OR: 0.96, CI: 0.92-0.99].

**Table 3:** Estimates from logistic regression analysis for low birth weight children by their background characteristics, India, 2015–16

Background characteristics	OR [95% CI]
**Age at index birth (in Years)**	
≤ 19	Ref.
20–24	0.82***(0.79–0.86)
25–29	0.77***(0.73–0.81)
30–35	0.75***(0.71–0.80)
35+	0.77**(0.70–0.85)
**Body mass index**	
Underweight	1.27***(1.23–1.32)
Normal	Ref.
Overweight and Obese	0.93***(0.89–0.97)
**Ante-natal care**	
No	Ref.
Partial	0.75***(0.71–0.8)
Full^a^	0.64***(0.60–0.68)
**Birth order and interval**	
First	Ref.
2–3 & < 24 months	0.89***(0.85–0.93)
2–3 & > 23 months	0.84***(0.81–0.88)
4+ & < 24 months	0.95(0.87–1.04)
4+ & > 23 months	0.81***(0.76–0.86)
**Education**	
No education	Ref.
Primary	1.04(0.99–1.09)
Secondary	0.91***(0.87–0.95)
Higher	0.73***(0.69–0.79)
**Caste**	
Scheduled Caste	1.06**(1.01–1.11)
Scheduled Tribe	0.93***(0.88–0.98)
Other Backward Class	0.97(0.93–1.01)
Others	Ref.
**Religion**	
Hindu	Ref.
Muslim	0.94**(0.90–0.99)
Others	0.79***(0.74–0.84)
**Wealth index**	
Poorest	Ref.
Poorer	0.95**(0.90–0.99)
Middle	0.91***(0.86–0.96)
Richer	0.91**(0.85–0.98)
Richest	0.79***(0.73–0.85)
**Residence**	
Urban	Ref.
Rural	0.89***(0.86–0.93)
**Sex of the child**	
Male	Ref.
Female	1.23***(1.19–1.26)
**Region**	
North	Ref.
Central	0.92***(0.88–0.97)
East	0.67***(0.64–0.71)
Northeast	0.51***(0.47–0.54)
West	0.94**(0.88–0.99)
South	0.84***(0.79–0.89)
**Source of drinking Water**	
Not improved	Ref.
Improved^b^	1.03(0.99–1.07)
**Type of toilet facility**	
Not improved	Ref.
Improved^c^	1.00(0.96–1.05)
**Type of cooking fuel**	
Unclean	Ref.
Clean^d^	0.96*(0.92–0.99)

*Ref.* Reference category, *OR* Odds ratio, *CI* Confidence interval

***p < 0.01; **p < 0.05; *p < 0.10

^a^Full ANC defined as women who received four or more antenatal checks, at least two tetanus toxoid injection, and took iron and folic acid tablets or syrup for 100 days or more during their last live birth in the 5 years preceding the survey

^b^Include piped water, public taps, standpipes, tube wells, boreholes, protected dug wells and springs, rainwater, and community reverse osmosis (RO) plants

^c^Include flush/pour flush toilets to piped sewer systems, septic tanks, and pit latrines; ventilated improved pit (VIP)/biogas latrines; pit latrines with slabs; and twin pit/composting toilets

^d^Include Electricity, LPG/natural gas, biogas

**Fig. 1:**
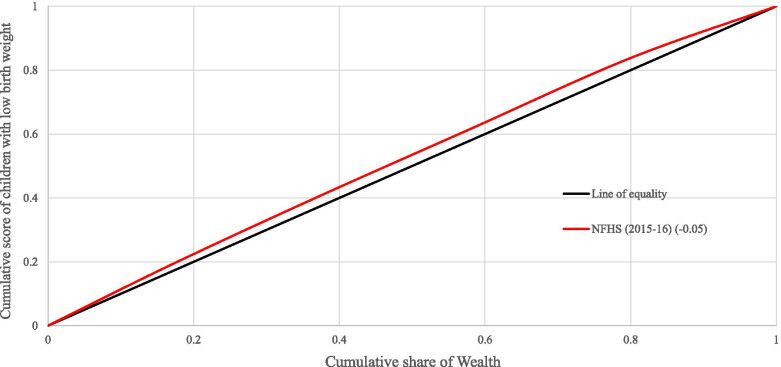
Concenrtration curve for children with low birth weight in India, 2015–16

Figure 1 depicts the concentration curve for children with LBW in India. The curve above the line of equality shows that LBW was concentrated among children from low socio-economic status. The negative value of the concentration index depicts that the outcome variable (LBW here) is concentrated among the poor. The value of CCI for India was (− 0.05).

**Fig. 2:**
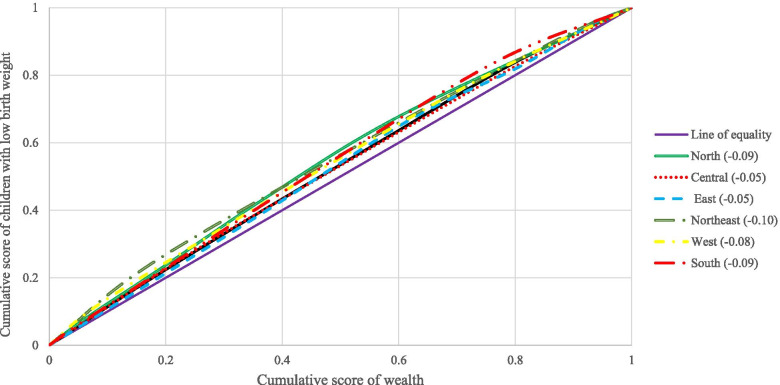
Concenrtration curve for children with low birth weight acorss six regions of India, 2015–16

Figure 2 depicts the concentration curve for children with LBW across six regions of India. The highest CCI was for the north-eastern region (− 0.10), followed by the southern region (− 0.09) and northern region (− 0.09) of India while the lowest CCI was for central (− 0.05) and east (− 0.05) regions.

Estimates of decomposition analysis for the contribution of various explanatory variables for LBW children in India is presented in Table 4. It was found that the wealth quintile explained 73.6% of the SES related inequality followed by the region of India (− 44%) and educational status (43.4%) for LBW among children in India. Additionally, BMI of the women (28.4%), ANC (20.8%), and residential status (− 15.7%) explained SES related inequality for LBW among children in India.

**Table 4:** Estimates of decomposition analysis for the contribution of various explanatory variables for low birth weight children in India, 2015–16

Background characteristics	Coefficient	Elasticity	CCI	Absolute Contribution to CCI	Percentage contribution
**Age at index birth**					
≤ 19 **(Ref.)**					
20–24	− 0.196***	− 0.009	0.004	0.000	0.4
25–29	− 0.264***	− 0.003	0.073	0.000	2.6
30–35	−0.286***	− 0.001	0.024	0.000	0.1
35+	− 0.261**	− 0.001	0.024	0.000	0.1
**Body mass index**					
Underweight	0.239***	0.009	−0.219	− 0.002	21.9
Normal **(Ref.)**					
Overweight and Obese	−0.072***	− 0.002	0.330	− 0.001	6.4
**ANC**					
No **(Ref.)**					
Partial	−0.285***	− 0.031	− 0.056	0.002	−18.6
Full	−0.446***	− 0.016	0.231	− 0.004	39.4
**Birth order and interval**					
First **(Ref.)**					
2–3 & < 24 months	−0.118***	−0.002	− 0.046	0.000	−1.1
2–3 & > 23 months	− 0.170***	−0.010	0.024	0.000	2.5
4+ & < 24 months	−0.050	0.000	−0.397	0.000	−1.3
4+ & > 23 months	−0.209***	−0.003	− 0.374	0.001	−11.6
**Education**					
No education **(Ref.)**					
Primary	0.036	0.001	−0.273	0.000	3.5
Secondary	−0.094***	−0.005	0.091	0.000	5.1
Higher	−0.308***	−0.006	0.579	−0.003	34.8
**Caste**					
Scheduled Caste	0.054**	0.000	−0.158	0.000	0.5
Scheduled Tribe	−0.077***	0.001	−0.396	0.000	2.1
Other Backward Class	−0.030	−0.002	0.036	0.000	0.6
Others **(Ref.)**					
**Religion**					
Hindu **(Ref.)**					
Muslim	−0.058**	−0.002	0.036	0.000	0.6
Others	−0.233***	−0.001	0.221	0.000	1.4
**Wealth index**					
Poorest **(Ref.)**					
Poorer	−0.056**	−0.002	−0.463	0.001	−8.5
Middle	−0.093***	−0.003	− 0.055	0.000	−1.7
Richer	−0.089**	−0.003	0.376	−0.001	10.1
Richest	−0.240***	−0.009	0.798	−0.007	73.7
**Residence**					
Urban **(Ref.)**					
Rural	−0.114***	−0.007	− 0.212	0.001	−15.7
**Sex of the child**					
Male **(Ref.)**					
Female	0.204***	0.013	−0.002	0.000	0.3
**Region**					
North **(Ref.)**					
Central	−0.080***	−0.004	−0.107	0.000	−4.5
East	−0.394***	−0.015	− 0.340	0.005	−56.2
Northeast	−0.682***	−0.003	− 0.220	0.001	−7.1
West	−0.066**	−0.003	0.158	−0.001	5.8
South	−0.175***	−0.008	0.202	−0.002	18.0
**Source of drinking Water**					
Not improved **(Ref.)**					
Improved^b^	0.030	−0.002	0.005	0.000	0.1
**Type of toilet facility**					
Not improved **(Ref.)**					
Improved^c^	0.003	−0.001	0.321	0.000	4.8
**Type of cooking fuel**					
Unclean **(Ref.)**					
Clean^d^	−0.039*	0.002	0.472	0.001	−8.1
**Calculated CCI**				−0.009	
**Actual CCI**				−0.056	
**Residual**				−0.047	

*Ref.* Reference category, *CCI* Concentration index

***p < 0.01; **p < 0.05; *p < 0.10

^a^Full ANC defined as women who received four or more antenatal checks, at least two tetanus toxoid injection, and took iron and folic acid tablets or syrup for 100 days or more during their last live birth in the 5 years preceding the survey

^b^Include piped water, public taps, standpipes, tube wells, boreholes, protected dug wells and springs, rainwater, and community reverse osmosis (RO) plants

^c^Include flush/pour flush toilets to piped sewer systems, septic tanks, and pit latrines; ventilated improved pit (VIP)/biogas latrines; pit latrines with slabs; and twin pit/composting toilets

^d^Include Electricity, LPG/natural gas, biogas

## Discussion

In the present paper, we have tried to identify the determinants of a child’s LBW by analyzing the fourth round of the National Family Health Survey. We found that LBW of a child was due to the following reasons: (i) Mother’s individual characteristics such as her age (below 19 years), low BMI status, with no ANC services and education; (2) Mother’s household characteristic such as if she belonged to the poorest wealth quintile, and scheduled caste; (3) If the child was a female and from a higher birth order & interval. We also found that the LBW of the child was concentrated in the poorest sections of society. Further, the mother’s wealth quintile, region and educational status contributed to the maximum while explaining the socio-economic related inequality for LBW among the children in India.

The present study results indicate that women who had low BMI status were more likely to have children with LBW status. This observation is in accordance with a study that shows that girls with low birth weight are likely to give birth to LBW infants [37]. Further, a strong positive relationship is found between the mother’s BMI and the child’s birth weight [38]. For instance, a secondary analysis of the second round of the National Family Health Survey found that mothers who were underweight were 30% more likely to have an LBW baby than those women who weighed normal or overweight [25]. The same study also found that mothers’ non-use of ANC services increased the risk of LBW of the child. Similar results have been described in other studies [39, 40]. The study results reveal that the LBW of a child significantly depends if the mother is an adolescent, illiterate and if the child is female; these observations are inconsistent with other studies [25, 39–45]. A cross-sectional study showed that pregnancy during the mother’s adolescence was associated with unfavorable outcomes such as LBW of the infants; however, it was linked to social vulnerability since it was only observed among adolescent mothers without a partner [46]. Often a positive relationship between a mother’s education and the child’s health is noticed [47]. This may be because educated mothers are aware of newborn care practices, health facilities and ANC services. However, it is argued that illiterate women are less likely to use maternal health care services for delivery assistance [48].

Our study found that female infants had a higher likelihood of being born LBW. Though the study finding is similar to few studies [45, 49], it contradicts another finding [50]. Results revealed if a child is of fourth birth order, then the likelihood of LBW declines. We found few studies that suggested LBW was common among first-order babies [51, 52] and if the child is of second and higher birth order, then the odds of LBW are low [45]. Results of bivariate and multivariate techniques used in the study indicate that belonging to the poorest wealth quintile is a critical determinant of a child’s LBW. For instance, women belonging to low socio-economic status are at a greater risk of being underweight [53], which eventually leads to giving birth to an LBW child. Few other studies showed that the LBW of a child is determined by the mother’s economic class [40, 44, 45]. Again, the LBW of a child decreased if the household used clean cooking fuel and improved toilet facilities. It is in line with previous studies [25, 43, 54]. Poor sanitation practices increase the odds of LBW newborns [51]. This shows how a woman’s nutrition, use of ANC services, environmental factors and infections are influenced by various socio-economic factors like food security, poverty, and women’s status [25]. Perhaps, the above findings highlight the importance of the woman’s individual or household’s financial status that influences access to improved toilet facilities, clean cooking fuel and better nutritional status, which are all necessary determinants to avoid giving birth to an LBW newborn.

The socio-economic inequality in LBW newborns in India was measured by Concentration Index and Concentration curve. The analysis showed that LBW is mostly concentrated in the North-eastern regions of the country, followed by the South and Northern parts of the country. However, a study shows that the Northern states of India had the highest concentration of LBW children [5]. Literary evidence reveals that pregnant women suffering from anaemia are most likely to give birth to LBW babies [55, 56]. Since the prevalence of anaemia in the north-eastern states is quite high [57, 58], it could be a possible explanation for such a result. Further, poor public health system [59] and low coverage of reproductive, maternal, neonatal and child health interventions in the north-eastern states [47, 60] are few other justifications for the high concentration of LBW in those regions of the country.

## Limitation

The present study uses a nationally representative recently published sample of a well-known large-scale survey in India. Therefore, the study results can be generalised well. However, we acknowledge some limitations too. First, because of the cross-sectional nature of the data, we could not draw any causal relationship between the variables. Second, another limitation of the cross-sectional household survey is that birth weight is estimated from the records of the newborn in health facilities. However, the birth weight of those born at home are not available.

## Conclusion

Since India contributes to the highest infant LBW in the southern region, identifying the risk factors of LBW has significant policy implications. Adequate attention should be given to the mother’s nutritional status; timely intake of iron and folic acid tablets during pregnancy can be a way of achieving it. Awareness of health education and usage of health services during pregnancy are few other important things that can be taken up at the household and community level. Further, there is a need to improve the coverage and awareness of the ANC program. Hence, the role of the health workers is of utmost importance. Programs on maternal health services can be merged with maternal nutrition to bring about an overall decline in the LBW of children in India.

## Supplementary Information

**Additional file 1: Table S1.** STROBE Flow Diagram.

## Data Availability

The study utilizes secondary source of data which is freely available in public domain through https://dhsprogram.com/methodology/survey-search.cfm?pgtype=main&SrvyTp=country&ctry_id=57.

## Abbreviations

CC: Concentration curve
CI: Confidence interval
CCI: Concentration index
NFHS: National Family Health Survey
LBW: Low birth weight
ANC: Ante-natal care
OR: Odds Ratio
BMI: Body mass index
UNICEF: United Nations International Children’s Emergency Fund
WHO: World Health Organization
