# Supplementary material for: Newborn low birth weight: do socio-economic inequality still persist in India?
Source: BMC Pediatr. 2021 Nov 19;21:518. doi: 10.1186/s12887-021-02988-3 (PMC8603541; doi:10.1186/s12887-021-02988-3)
Supplement: Supplementary file 1 — Additional file 1: Table S1. STROBE Flow Diagram. [file 12887_2021_2988_MOESM1_ESM.doc]

**Table-S1**: STROBE Flow Diagram

|  | Item No |  | | Recommendation |
| --- | --- | --- | --- | --- |
| **Title and abstract** | 1 |  | | Page 1-2 |
|  | | | Introduction | |
| Background/rationale | 2 |  | | Page 3-6 |
| Objectives | 3 |  | | Page 6 |
|  | | | Methods | |
| Study design | 4 |  | | Page 7 |
| Setting | 5 |  | | Page 7 |
| Participants | 6 |  | | Page 7 |
| Variables | 7 |  | | Page 8-9 |
| Data sources/ measurement | 8* |  | | n/a |
| Bias | 9 |  | | n/a |
| Study size | 10 |  | | Page 7 |
| Quantitative variables | 11 |  | | n/a |
| Statistical methods | 12 |  | | Page 9-12 |
|  | | | Results | |
| Participants | 13* |  | | n/a |
| Descriptive data | 14* |  | | n/a |
| Outcome data | 15* |  | | n/a |
| Main results | 16 |  | | Page 12-14 |
| Other analyses | 17 |  | | n/a |
|  | | | Discussion | |
| Key results | 18 |  | | Page 15 |
| Limitations | 19 |  | | Page 17 |
| Interpretation | 20 |  | | Page 15-17 |
| Generalisability | 21 |  | | Page 17 |
|  | | | Other information | |
| Funding | 22 |  | | Page 19 |

*Give information separately for exposed and unexposed groups.
